# A new perspective on semen quality of aged male: The characteristics of metabolomics and proteomics

**DOI:** 10.3389/fendo.2022.1058250

**Published:** 2023-01-04

**Authors:** Yi Guo, Jinli Li, Fengdan Hao, Yang Yang, Hao Yang, Qiurong Chang, Pengcheng Kong, Wenqiang Liu, Xianting Jiao, Xiaoming Teng

**Affiliations:** ^1^ Department of Assisted Reproduction, Shanghai First Maternity and Infant Hospital, School of Medicine, Tongji University, Shanghai, China; ^2^ Department of Pediatrics, Tongji Hospital, School of Medicine, Tongji University, Shanghai, China; ^3^ Department of Pediatric Cardiology, Xinhua Hospital Affiliated to Shanghai Jiao Tong University School of Medicine, Shanghai, China

**Keywords:** advanced male age, metabolome, proteome, sperm quality, biomarker

## Abstract

**Background:**

Semen quality is negatively correlated with male age and is mainly quantified by a routine semen analysis, which is descriptive and inconclusive. Sperm proteins or semen metabolites are used as the intermediate or end-products, reflecting changes in semen quality, and hold much promise as a new biomarker to predict fertility in advanced-aged males.

**Objectives:**

In this study, we sought to assess whether the semen metabolome and proteome of aged males can affect semen quality and serve as biomarkers for predicting semen quality.

**Materials and methods:**

We retrospectively analyzed 12825 males that underwent semen routine analysis to understand the age-dependent changes in sperm quality. To identify the difference between aged and young adults, metabolomics (n=60) analyses of semen and proteomics (n=12) analyses of sperm were conducted. Finally, integrated machine learning of metabolomics was conducted to screen biomarkers to identify aging semen.

**Results:**

We discovered that male age was positively correlated with sperm concentration as well as DNA fragmentation index(DFI), and negatively with progressive motile sperm count, total sperm count, sperm volume and progressive sperm motility. The differential metabolites were significantly enriched in various metabolic pathways, and four of these differential metabolites (Pipamperone, 2,2-Bis(hydroxymethyl)-2,2’,2’’-nitrilotriethanol, Arg-Pro and Triethyl phosphate) were utilized to establish a biomarker panel to identify aging semen. Proteomic analysis showed that differential proteins were significantly enriched in protein digestion and absorption and some energy-related pathways. An integrated analysis of the metabolome and proteome identified differential energy metabolism and oxidative stress-related proteins, which could explain the decreased motility and the increased DFI of aging sperm

**Discussion and conclusion:**

We provide compelling evidence that the changes in semen metabolome and sperm proteome are related to the decline of semen quality in aged males. Moreover, a biomarker panel based on four metabolites was established to identify aging semen.

## 1 Introduction

It is well-established that advanced maternal age is related to lower fertility and birth defects (1). Growing evidence substantiates that advanced male age exerts deleterious effects on semen quality, assisted reproductive outcomes, and offspring health (2). In this regard, advanced paternal age has been related to a higher risk of diseases in offspring, including achondroplasia, autism, schizophrenia, bipolar disorders, cancers and impaired cognitive abilities (3). Given that the exact mechanisms remain largely unknown, exploring the biological characteristics of aging semen and finding explanations for the decline of fertility and offspring health of advanced males is essential.

Routine semen analysis remains the main indicator for evaluating semen quality, including semen volume, pH, sperm count, motility, vitality, and morphology (4). In recent years, researchers have made vast strides with the discovery of more advanced semen tests to evaluate sperm function, including oxidative stress and DNA fragmentation index (DFI), for further evaluation of sperm function. Although semen analyses have identified several different parameters with different predictive values for sperm function, these parameters are often descriptive and can not provide a more etiological explanation. Accordingly, the application of routine semen analysis to diagnose male fertility has been controversial for many years (5, 6). In recent years, omics, including genomic, proteomic and metabolomic techniques, have been used to analyze molecular-level information of samples to diagnose or treat male infertility (7). Indeed, in-depth analyses of sperm molecular composition and data mining have contributed to our understanding of sperm function. A previous study identified 103 sperm proteins that play a key role in the fertilization, including sperm penetration, sperm acrosome reaction, and sperm-oocyte hybridization (8). Meanwhile, a comparative proteome analysis between asthenozoospermia and normozoospermia yielded eight differentially expressed proteins (DEPs), mainly related to energy and metabolism (9). More interestingly, one sperm protein CATSPER1, the sperm-specific calcium channel, may be considered as a valuable tool to predict ART success (10). These DEPs are potentially more effective and reliable indicators to evaluate male fertility with respect to sperm function and fertilization. On the other hand, metabolites are thought to be the end products of cellular regulatory processes and biological systems following genetic or environmental changes (11). Compared with genes and proteins, endogenous metabolites are the closest to the phenotype and more easily detected (11). The seminal metabolome has recently become a research hotpot and is considered as a promising tool to uncover biomarkers of semen quality and fertilizing capacity (12). It has been found that 63 metabolites are potential biomarkers of male infertility, of which 17 are correlated with semen parameters (13). Reduced levels of metabolites in seminal plasma, including amino acids, lactate, citrate, creatinine, α-ketoglutaric acid, spermine and putrescine, have also been used to distinguish oligoasthenoteratozoospermia from normozoospermic controls (14). In addition, the metabolic profile of seminal plasma was used to predict spermatogenesis in nonobstructive azoospermic (NOA) patients in assisted reproduction (15). Taken together, the above findings suggest that applying proteomics and metabolomics in andrology can help overcome the limitations of conventional semen analysis.

It is widely acknowledged that aging affects a myriad of genetic, biochemical, and metabolic processes (16). However, no study has hitherto reported the effect of aging on semen proteomics and metabolomics. It remains unclear whether proteomics and metabolomics analyses can explain the decline of semen quality in aged males. Because semen is mainly composed of secretions from accessory glands, it contains a large number of metabolites, and is easier to be detected for being biomarkers. In addition, in the process of sperm-oocyte binding, sperm proteins are more involved in the fertilization and suitable for exploring sperm function. Herein, we sought to establish the profiles of semen metabolome and sperm proteome to identify age-related changes in metabolites and proteins between aged and young men. Meanwhile, we established a biomarker panel to predict aging semen.

## 2 Material and methods

### 2.1 Study design and patients

We retrospectively analyzed the data of 12828 sperm samples collected from males that underwent routine semen examinations and DFI tests from January 2020 to December 2021 at the Department of Assisted Reproduction of Shanghai First Maternity and Infant Hospital. All participants were categorized into five groups according to their age as follows: group A (≤30y), group B (31-35y), group C (36-40y), group D (41-45y) and group E (≥46y). Patients were excluded for the following reasons: history of sexually transmitted diseases, chromosome abnormalities, mumps orchitis,varicocele, testicular surgery, epididymal surgery, urethral surgery and vasectomy.

**Figure 1:**
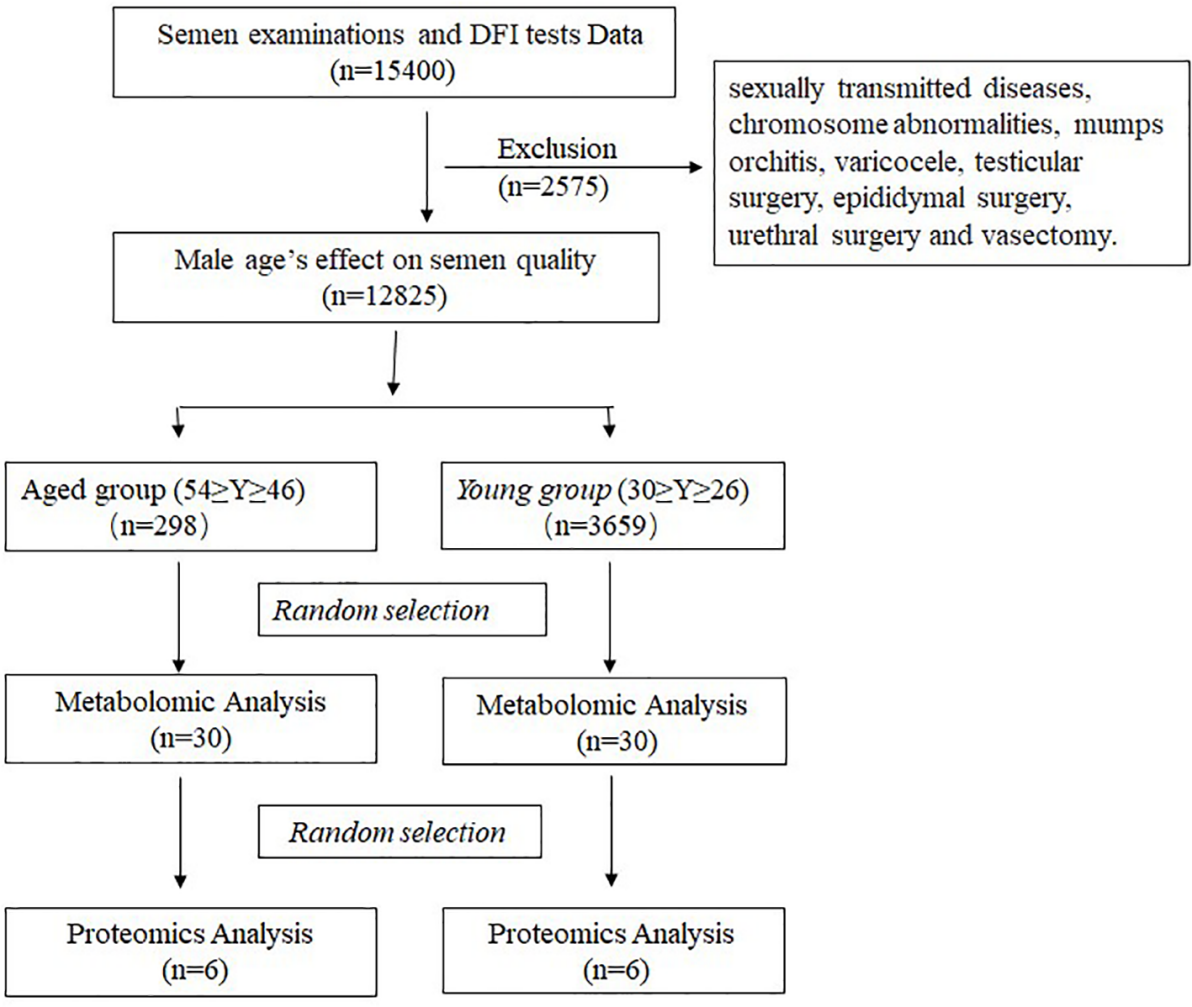
Study design. Flowchart of the research design.

60 participants were enrolled in the metabolomics analysis, of which 12 participants were also enrolled in the proteomic analysis. Participants aged 45-54 were included in the aged semen (AS) group, while participants aged 26-30 comprised the young semen (YS) group. The exclusion criteria were as follow: 1) metabolic diseases (diabetes and cardiovascular diseases); 2) long-term exposure to toxins, tobacco, alcohol or drugs; 3) other known causes male infertility (genetic disease and infection). The Shanghai First Maternity and Infant Hospital’s medical ethics committee approved the study, and all participants provided written informed consent. The study design is illustrated in **Figure 1**.

### 2.2 Semen collection, analysis and purification

Semen samples were obtained by masturbation after 2-7 days of abstinence. After 30 minutes of liquefaction in an incubator (37°C), the following parameters, including semen volume, pH, sperm concentration, motility, total sperm count and progressive motile sperm count, were evaluated according to the World Health Organization guidelines. Semen volume was measured by weighing, and sperm motility and concentration were analyzed by computer-assisted sperm analysis (CASA) (Hamilton Thorne, USA). At the same time, part of the semen samples were used for DFI testing and the detection of sperm DFI was performed according to the kit’s instructions (CellPro Biotech, China), which was based on the sperm chromatin structure assay (SCSA) method.

Sperm were isolated by the density gradient method. In brief, 2–3 mL of liquefied semen samples was carefully overlaid onto a two-layer (40–80%) Percoll gradient (1:1:1, v/v/v). After centrifugation at 300×g for 15 mins, the supernatant was discarded and the sperm pellet was washed twice with PBS. Then the purified sperm was examined under a microscope(Olympus, Japan) to ensure no lymphocytes, epithelial cells and bacteria.

### 2.2 Metabolomic analysis

For metabolite extraction,1mL of metabolite extraction buffer consisting of methanol/acetonitrile/H2O (2:2:1,v/v/v) was added to the semen sample and vortexed. The mixture was collected into a 1.5mL centrifuge tube and centrifuged at 14000×g for 10 min to remove proteins. The supernatant was dried up and re-dissolved in a solvent containing acetonitrile/water (1:1, v/v), and then separated using liquid chromatography, UHPLC (1290 Infinity LC, Agilent Technologies) and subjected to quadrupole time-of-flight mass spectrometry (AB Sciex TripleTOF 6600). Briefly, a 2.1 mm × 100 mm ACQUIY UPLC BEH 1.7 µm column (Waters, Ireland) was used to separate the sample solution. Meanwhile, the mobile phase in both ESI positive and negative modes contained A=25 mM ammonium acetate and 25 mM ammonium hydroxide in water and B= acetonitrile. The gradient was as follows: 0–0.5 min, 95% B; 0.5–7min, 95%–65% B; 7–8min, 65%–40% B; 8–9min, 40% B; 9–9.1min, 40%–95% B; 9.1–12min, 95% B. A random injection sequence was employed to avoid signal fluctuations during sample analysis. The quality control (QC) samples were inserted to ensure a reliable data analysis. In MS-only acquisition, the m/z range was set to 60~1000 Da, and the accumulation time for the TOF MS scan was set to 0.20 s/spectra. In the auto MS/MS acquisition, the m/z range was set to 25~1000 Da, and the accumulation time for the product ion scan was set to 0.05 s/spectra. The information-dependent acquisition (IDA) mode was used to acquire the product ion scan. The following parameters were set: the collision energy (CE) was set at 35 V ± 15 eV; declustering potential (DP) was set to ±60 V ( ± ESI); isotopes within 4 Da were excluded and 10 candidate ions were monitored per cycle. Metabolomic Analysis was conducted by Shanghai Applied Protein Technology Co.

Only variables with more than 50% nonzero measurements in one or more groups were retained during the extraction of ion features in the metabolome. Metabolite analysis was carried out by comparing their m/z accuracy (<25 ppm) and MS/MS spectra to an in-house database based on well-established standards. After sum normalization, an R package was used to perform orthogonal partial least-squares discriminant analysis (OPLS-DA). Pearson’s correlation analysis was used to determine the relationship between two variables. The KEGG database was used for pathway enrichment analysis (http://geneontology.org/).

### 2.3 Proteomics analysis

Sperm samples (six replicates for each group) were isolated by the density gradient method and washed twice with PBS for the proteomics study. 2×10^6^ sperm for each sample were lysed by 200μL SDT buffer (3×volume of sperm pellet) containing 4% SDS, 150 mM Tris-HCl pH 8.0 and 100 mM DTT. The lysates were further sonicated and boiled for 10 min. After centrifugation at 14000g for 30 min at 4°C, the supernatant was quantified with the BCA Protein Assay Kit (Bio-Rad, USA). 15μg protein from each sample was individually separated by one-dimensional (1D) Sodium dodecyl sulfate (SDS) Polyacrylamide gel electrophoresis (PAGE) and then individually digested using in-gel trypsin for sample preparation. An equal aliquot of each sample was pooled, and the mixture was used for generating the DDA library. For the pool sample adhesive strip, the high-abundance strip (45KD-66.2KD) was cut as the high-abundance sample, and the remaining part regarded as the low-abundance sample, underwent in-gel trypsin method. The peptides from each sample were desalted using C18 Cartridges (Empore™ SPE Cartridges, Sigma). The peptides were estimated by UV light spectral density at 280 nm using an extinction coefficient of 1.1 of 0.1% (g/l) solution. The peptides of low-abundance sample were fractionated into ten fractions using the Thermo Scientific Pierce High pH Reversed-Phase Peptide Fractionation Kit. Each fraction was concentrated by vacuum centrifugation and reconstituted in 15µl of 0.1% (v/v) formic acid. Collected peptides were desalted on C18 Cartridges (Empore™ SPE Cartridges C18 (standard density), bed I.D. 7 mm, volume 3 ml, Sigma) and reconstituted in 40µl of 0.1% (v/v) formic acid. The iRT-Kits(Biognosys) wer added to correct the relative retention time differences between runs with volume proportion1:3 for iRT standard peptides versus sample peptides.

To create the query database for data-independent acquisition (DIA) mass spectra analysis, a spectral library was built using data-dependent acquisitions (DDA). The Q Exactive 480 mass spectrometer connected to a Thermo Scientific Easy nLC 1200 chromatography system was used to perform MS on all fractions for DDA library generation. First, the high-abundance sample and all fractions (from the low-abundance sample) were loaded onto a Thermo Scientific EASY-Spray TM C18 Trap column with the following specifics and dimensions: P/N 164946, 3 um, 75 um*2 cm. It was then separated using a Thermo Scientific EASY-SprayTM C18 LC Analytical Column with the following parameters: ES802, 2 um, 75 um*25 cm. The full-scan MS detection was conducted in the positive ion mode, with the scan range set to 300~1800 m/z. The resolution was 60000 at 200 m/z for MS1 scans and 15,000 for sequential MS2 scans.

LC-MS/MS set to the DIA mode was used to assess the peptides from each sample by Shanghai Applied Protein Technology. For DIA mass spectrometry, 2 μg peptides from each sample were mixed with iRT peptide before injection. Chromatographic separation was performed with the Easy nLC-1200, and the Q-Exactive 480 mass spectrometer was used for automatic switching between MS and MS/MS acquisition. In each DIA cycle, one full MS–SIM scan was performed, and 30 DIA scans were performed utilizing a mass range of 350–1800 m/z with the following settings: SIM full scan resolution was set to 120,000 at 200 m/z; AGC 3e6; a maximum IT 50ms; profile mode; DIA scans were performed at a resolution of 15,000; AGC target 3e6; Max IT auto; and the normalized collision energy used was 30 eV. QC samples were injected with DIA mode at the start of MS to monitor performance, and QC was performed every five injections throughout the experiment. Quantitative Proteomics Analysis was conducted by Shanghai Applied Protein Technology Co.

For DDA library data, the FASTA sequence database (http://www.uniprot.org.) was searched with SpectronautTM 14.4.200727.47784 (Biognosys) software. The iRT peptides sequence was added, and the parameters were set as follows: enzyme: trypsin, max missed cleavages: 2, fixed modification: carbamidomethyl(C), dynamic modification: oxidation(M) and acetyl (Protein N-term). All reported data were based on 99% confidence for protein identification as determined by false discovery rate (FDR =N(decoy)×2/(N(decoy)+ N(target))) ≤ 1%. The spectral library was constructed by importing the original raw files and DDA searching results into Spectronaut Pulsar X TM_12.0.20491.4 (Biognosys). DIA data was analyzed with SpectronautTM 14.4.200727.47784, searching the above constructed spectral library. The main software parameters were as follows: retention time prediction type: dynamic iRT, and interference on MS2 level correction and cross-run normalization were enabled. All results were filtered based on a Q cutoff value of 0.01 (equivalent to FDR<1%).

The protein differences were calculated using Fisher’s exact test, and a P-value<0.05 was statistically significant. Subsequently, we conducted gene ontology (GO) analysis on the DEPs using Blast2GO (https://www.blast2go.com/) and KEGG Orthology analysis using the KEGG database. Protein-protein interaction (PPI) networks were constructed using the STRING database (http://string-db.org/) to determine the functional relationship between DEPs.

### 2.4 Integrated machine learning

Each metabolite’s comprehensive weight value was calculated, and ROC analysis was used to assess the effect of each metabolite on the area under the receiver operating characteristic curve (AUC) values. The candidate biomarker panel comprised the top-ranked metabolites with the highest AUC value. To validate candidate biomarkers, integrated analyses such as Logistic Regression (LR), Random Forest (RF), and Support Vector Machine (SVM) were used. Subsequently, Logical Regression was used to construct the classification model, and the entire dataset was randomly divided into training and test sets in a 7:3 ratio. Notably, the training set was utilized for hyper-parameter optimization and model training, while the test set was used to validate of the model.

### 2.5 Statistical analysis

Multivariable linear regression models with adjustment were used to investigate the relationship between male age and sperm parameters. Statistical analysis was conducted using Stata 12.0 and significant difference were indicated as follows: *P < 0.05, **P < 0.01 and ***P < 0.001.

### 2.6 Large-scale data

The raw metabolomics data were uploaded to MetaboLights at EMBL-EBI under accession number MTBLS5289 and the raw proteomics data were uploaded to the ProteomeXchange Consortium (http://proteomecentral.proteomexchange.org) under accession number PXD035466

## 3 Results

### 3.1 Male age’s effect on semen quality

**Figure 2:**
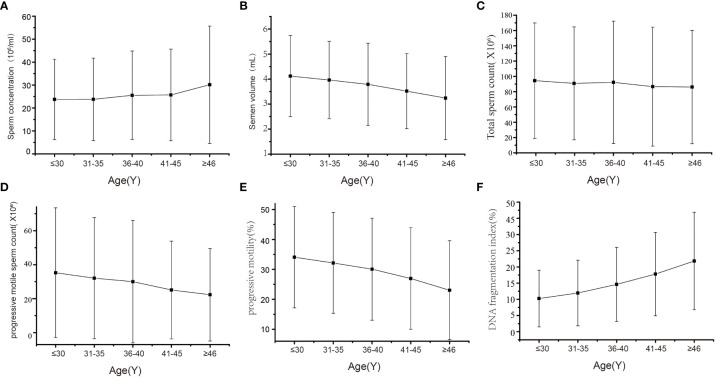
Trends in semen quality parameters with male ages. **(A)** Sperm concentration **(B)** Semen volume **(C)** Total sperm count **(D)** Progressive motile sperm count **(E)** Progressive motility **(F)** DNA fragmentation index.

Overall, a total of 12 825 semen samples were included in the present study. We calculated and recorded the semen pH, sperm concentration, volume, total sperm count, progressive motile sperm count, DFI and progressive sperm motility. The details of the semen parameters across age categories are shown in **Table 1**, and the trends are shown in **Figure 2**. After adjustment for abstinence time and semen pH, we evaluated the trends for the semen parameters with respect to age using multiple linear regression analyses. As shown in **Table 2**, the average semen volume decreased from 4.12 mL± 1.62 in group A to 3.24 mL± 1.66 in group E, with an average decline of 0.04 mL per year. The total sperm count was reduced from 94.35million ±75.59 to 86.14million ± 74.08, with an average annual decline of 0.56million. Moreover, the progressive motility was reduced from 34.07% ± 16.96 to 23.03% ± 16.51, with an average annual decline of 0.46%, and the progressive motile sperm count decreased from 35.27 million ± 38.18 to 22.32 million ± 27.21, with an average annual decline of 0.65 million. Finally, the sperm concentration increased from 23.75 million/mL ± 17.51 to 30.16 million/mL ± 25.57, with an average annual increment of 0.18 million/mL and sperm DFI increased from 10.24% ± 8.75 to 21.82% ± 15.05, with an average annual increment of 0.49%.

**Table 1:** The descriptive statistics of semen parameters and DFI by male age category.

Age group	A (≤30)	B (31-35)	C (36-40)	D (41-45)	E (≥46)
Number (%)	4638(36.16)	5020(39.14)	2094(16.33)	744(5.80)	329(2.57)
Abstinence time(days)	4.21 ± 2.87	4.29 ± 2.88	4.49 ± 3.24	4.71 ± 4.40	4.88 ± 3.29
pH	7.22 ± 0.18	7.22 ± 0.18	7.23 ± 0.19	7.23 ± 0.18	7.22 ± 0.20
Volume(ml)	4.12 ± 1.62	3.96 ± 1.55	3.79 ± 1.64	3.52 ± 1.50	3.24 ± 1.66
Sperm concentration(10^6^/ml)	23.75 ± 17.51	23.79 ± 17.96	25.55 ± 19.28	25.67 ± 19.98	30.16 ± 25.57
Total sperm count (10^6^)	94.35 ± 75.59	90.84 ± 73.78	92.19 ± 79.98	86.63 ± 77.74	86.14 ± 74.08
Progressive motility (%)	34.07 ± 16.96	32.15 ± 16.83	30.06 ± 17.03	26.95 ± 16.98	23.03 ± 16.51
Progressive motile sperm count (10^6^)	35.27 ± 38.18	32.03 ± 35.63	30.02 ± 36.00	25.12 ± 28.76	22.32 ± 27.21
DFI (%)	10.24 ± 8.75	11.94 ± 10.12	14.62 ± 11.43	17.79 ± 12.88	21.82 ± 15.05

Data are expressed as mean ± standard deviation. DFI DNA fragmentation index.

**Table 2:** Linear regression analyses of age trend in semen parameters.

Semen parameter	B (95% CI)	SE	t	P value
Volume (ml)	-0.04 (-0.05, -0.04)	0.00	-15.44	<0.001
Sperm concentration (10^6^/ml)	0.18 (0.11, 0.24)	0.03	5.70	<0.001
Total sperm number (10^6^)	-0.56 (-0.81, -0.32)	0.12	-4.55	<0.001
Progressive motility (%)	-0.46 (-0.52, -0.41)	0.03	-16.17	<0.001
Progressive motile sperm count (10^6^)	-0.65 (-0.77, -0.53)	0.06	-10.64	<0.001
DFI(%)	0.49 (0.46, 0.53)	0.17	28.78	<0.001

Noted: Adjustment for abstinence time and pH. CI, Confidence Interval; SE, Standard Error; DFI, DNA Fragmentation Index.

### 3.2 Metabolomics analysis of semen

**Figure 3:**
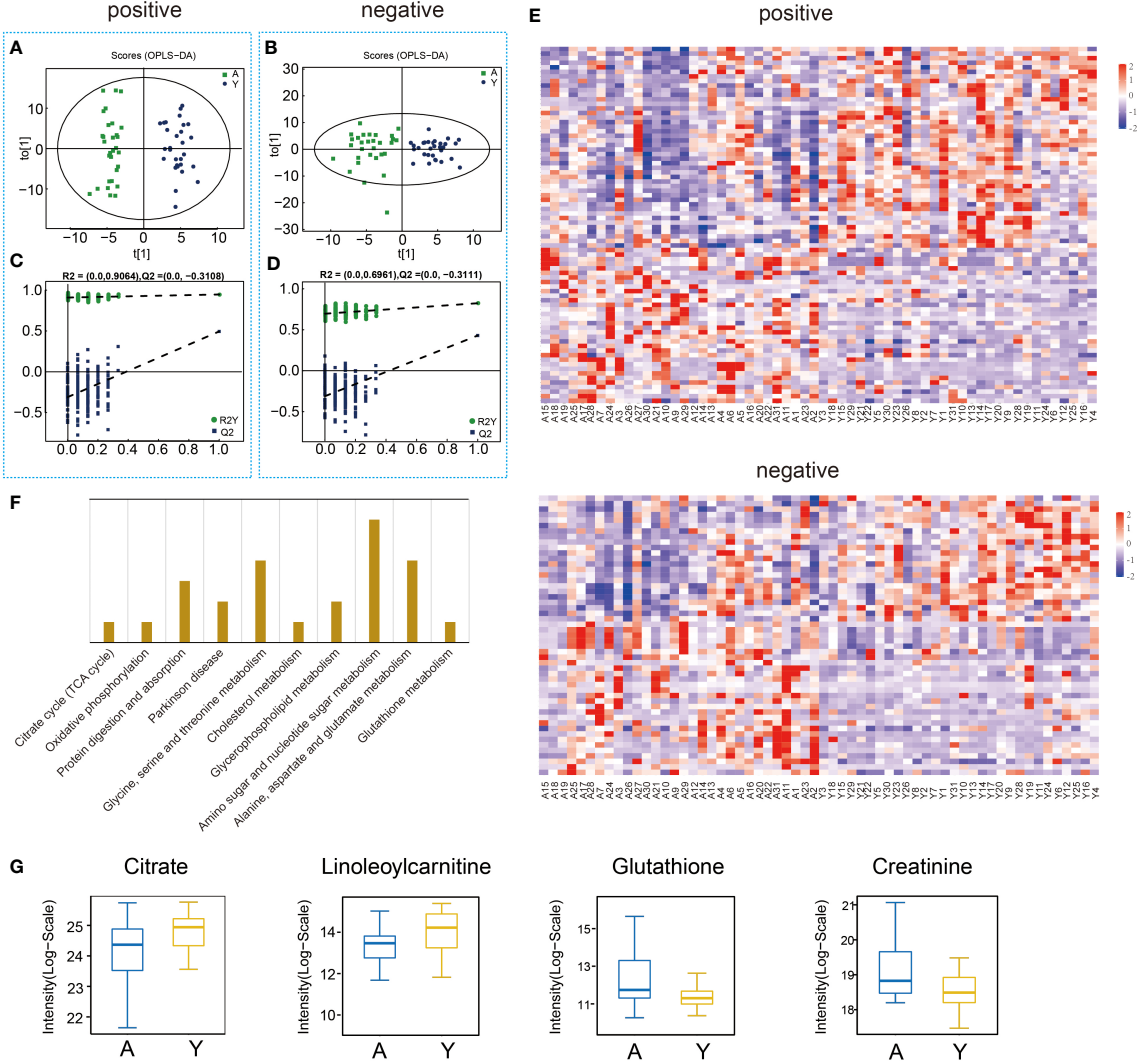
Semen sample metabolic profiling and analysis. **(A)** OPLS–DA models (ESI+) of UPLC-Q-TOF/MS metabonomic data for aged male (A) and young male (Y) groups. **(B)** OPLS–DA models (ESI-) of UPLC-Q-TOF/MS metabonomic data for groups A and Y. **(C)** Diagram of model verification in positive ion mode. **(D)** Diagram of model verification in negative ion mode. **(E)** Heat map for metabolites. **(F)** Enriched KEGG pathway between groups A and Y. **(G)** The differential expression of related metabolites in groups A and Y.

Thirty semen samples per group for aged and young participants were detected using ultra-high-performance liquid chromatography-quadrupole time-of-flight mass spectrometry (UPLC-Q-TOF/MS). 1331 positive-ion-mode and 870 negative-ion-mode metabolites were identified. The supervised OPLS-DA analysis revealed that the aged and young groups were completely divided into positive and negative ionization modes, which were validated by typical 7-fold cross-validation. Accordingly, high values were obtained for R2Y (0.824 and 0.944) and Q2 (0.430 and 0.494) parameters (**Figures 3A, B**). In addition, the 200 iterations permutation test was conducted to prevent overfitting of the OPLS-DA model (**Figures 3C, D**). 129 differentially expressed metabolites (62 downregulated and 67 upregulated) were identified following multivariate and univariate analyses based on the statistical significance criteria (VIP>1 and P<0.05). Significant differential metabolites are listed in **Table SI**, and the cluster heatmap of the significant differential metabolites is shown in **Figure 3E**. KEGG pathway analysis showed significant enrichment in a variety of metabolic pathways, including the citrate cycle (TCA cycle), Parkinson’s disease, cholesterol metabolism, glycerophospholipid metabolism, oxidative phosphorylation, amino acid metabolism, amino sugar and nucleotide sugar metabolism, glutathione metabolism, protein digestion and absorption pathways (**Figure 3F**). Results of quantitative analysis of important metabolites are shown in **Figure 3G**, which were mainly related to energy metabolism and oxidative stress.

### 3.3 Integrated machine learning of semen metabolites

**Figure 4:**
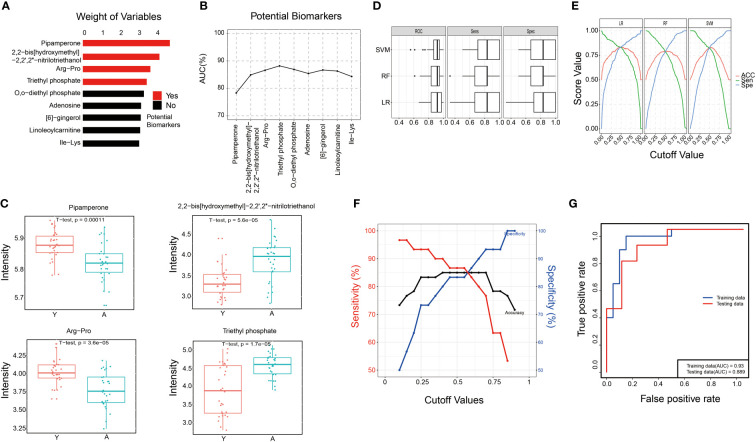
Integrated machine learning of semen metabolites. **(A)** The weight values of partial metabolites. **(B)** ROC analysis of each metabolite on AUC. **(C)** The relative concentrations of four metabolites in aged male (A) and young male (Y) groups. **(D, E)**. Verification of the four metabolites as the candidate biomarker by three different machine learning methods. **(F)** The optimal cutoff value was calculated by the Youden-index and the sensitivity, specificity, and accuracy of the diagnostic model. **(G)** Verification of the diagnostic panel model.

The comprehensive weight value of each metabolite was calculated, and the candidate biomarkers were sorted according to their weight values (**Figure 4A**). Then, we performed ROC analysis to evaluate the effect of each metabolite on AUC values and found that the top four metabolites (Pipamperone, 2,2-Bis(hydroxymethyl)-2,2’,2’’-nitrilotriethanol, Arg-Pro, Triethyl phosphate) exhibited the best performance for predicting decreased fertility (**Figure 4B**). The relative concentrations of these four metabolites in two groups were presented in **Figure 4C**, and Pearson’s correlation analysis of the candidate biomarkers was also performed to show the low correlation between these metabolites (**Figure S1**). To validate the effects of the four metabolites as the candidate biomarker, three different machine learning methods were used and compared, including Logistic Regression (LR), Random Forest (RF), and Support Vector Machine (SVM). The sensitivity, specificity, and accuracy of the three differential models were all greater than 0.8, which substantiated the good predictive performance of these four metabolites for aging semen (**Figures 4D, E**). At last, a diagnostic model was constructed by LR to distinguish aging semen from normal semen. The optimal cutoff value of the diagnostic model determined by the Youden-index was 0.58, with a sensitivity, specificity, and accuracy of 0.87, 0.87 and 0.87, respectively (**Figure 4F**). To test the diagnostic panel model, ROC analysis was applied to 70% of the metabonomic data to train the models, and the trained models were then used for prediction of the remaining 30% metabonomic data, which yielded an AUC value of 0.93 in the training set and 0.889 in the test set (**Figure 4G**).

### 3.4 Proteomic analysis of sperm

**Figure 5:**
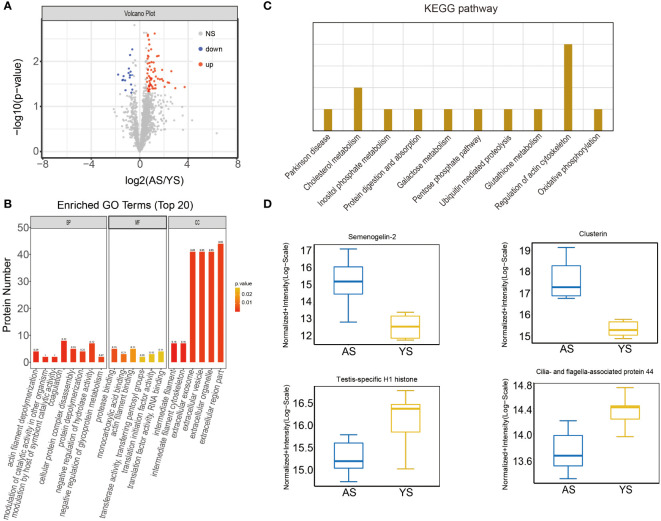
The proteomic profiles and protein analysis of sperm samples. **(A)** Volcano map for proteins. **(B)** Enriched GO terms between aged male (A) and young male (Y) group. **(C)** Enriched KEGG pathway between groups A and Y. **(D)** The differential expression of related proteins in groups A and Y.

A total of 2369 proteins were identified in both groups using the DIA quantitative proteomics approach, of which 80 proteins were significantly altered (fold change > 1.5 or < 0.67, p < 0.05) (**Table SII**). Specifically, 64 upregulated and 16 downregulated proteins were detected in the aged group compared to the young group (**Figure 5A**). GO analysis was conducted to identify the enriched GO terms of the DEPs. The top 20 GO terms are shown in **Figure 5B**, including actin filament depolymerization, cellular protein complex disassembly and protein depolymerization in biological processes, protease binding and actin filament binding in molecular functions in extracellular region part, intermediate filament and intermediate filament cytoskeleton in cellular components. KEGG analysis showed that DEPs were significantly enriched in protein digestion and absorption, galactose metabolism, pentose phosphate pathway, ubiquitin-mediated proteolysis, oxidative phosphorylation, glutathione metabolism, Parkinson’s disease, regulation of actin cytoskeleton, cholesterol metabolism and inositol phosphate metabolism (**Figure 5C**). The expression level of semenogelin-2, clusterin, testis-specific H1 histone and cilia- and flagella-associated protein 44 were significantly differentially expressed in the aged group (**Figure 5D**).

### 3.5 Integrated metabolomics and proteomics analysis

**Figure 6:**
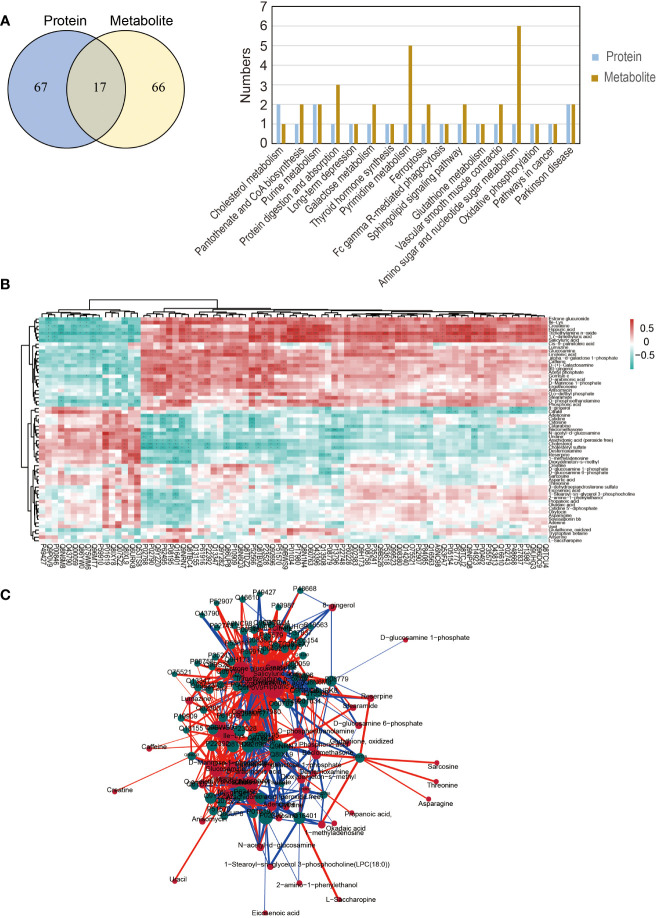
Integrated metabolomics and proteomics analysis. **(A)** The Venn diagram and common KEGG pathways of differential proteins and metabolites. **(B)** Pearson correlation hierarchical cluster analysis of differential proteins and metabolites. **(C)** The correlation network of differential proteins and metabolites.

An integrated analysis of differential metabolites and proteins in both groups was conducted to further evaluate the influence of male age on semen quality. Seventeen KEGG pathways were found to be involved in both metabolomics and proteomics, including oxidative phosphorylation, glutathione metabolism, protein digestion and absorption, galactose metabolism, cholesterol metabolism, Parkinson’s disease, pantothenate and CoA biosynthesis and the Sphingolipid signaling pathway (**Figure 6A**). Pearson correlation hierarchical cluster analysis was performed to objectively reflect the expression patterns of differential proteins and metabolites, and similar expression patterns involved in the similar biological process were grouped into the same cluster (**Figure 6B**). We then constructed a correlation network to assess the association between differential proteins and metabolites (**Figure 6C**).

## 4 Discussion

Our current study constructed complete metabolic profiles of semen and proteomic profiles of sperm from aged and young males and identified the differential metabolites and proteins to explain the age-dependent decline in semen quality. More importantly, we obtained a metabolite-related biomarker panel to assist in identifying aging semen.

It is widely acknowledged that continuous division of spermatogonial stem cells (SSCs) sustain spermatogenesis and fertility throughout male life (17). Advanced male age has become a potential risk factor for male infertility and offspring health (18, 19). The present study substantiated the significant influence of male age on all sperm parameters (except pH) and established a model predicting alterations in semen parameters with age using 5-year age bands. We found that male age was positively associated with sperm concentration and DFI but negatively associated with semen volume, total sperm count, progressive motility and progressive motile sperm count, which was consistent with a recent large retrospective study (20). Elevated sperm DFI with male age is well-recognized as an important indicator of sperm DNA damage. The decline of total sperm count and progressive motile sperm count is widely thought to significantly impair sperm fertilization capacity. In addition, we found that the sperm concentration increased while the semen volume decreased with age. We speculated that the decrease in semen volume might be due to the impaired function of the accessory glands of the male reproductive tract as a result of aging. Indeed, when the total sperm count did not change significantly, the decrease in semen volume would inevitably lead to an increase in sperm concentration. Albeit the mechanisms of age-dependent semen quality decline remain largely unclear, current evidence suggests that aging-induced oxidative stress could be a major cause. Accordingly, exploring the involved mechanisms and discovering the biomarkers of aging semen is paramount.

Semen reportedly contains many metabolites including nucleosides, lipids, monosaccharides, amino acids, minerals and electrolytes which are essential in regulating the motility and fertilization of sperm (21). As an easily accessible noninvasive material, semen provides a quick and objective assessment of dysregulated metabolism, suggesting it hold much promise as a new biomarker to predict fertility in advanced-aged males (22). In addition, the dysregulated sperm protein expression is significantly related to male infertility (23). In this study, we constructed the sperm proteomic and semen metabolomic profiles of aged and young males and then analyzed the potential functions of differential proteins and metabolites, which were helpful for further exploration of the mechanism involved. We analyzed sperm proteomics using DIA technology, which could record information on all ions for higher data coverage and improve the quantitative analysis efficiency of the identified proteins and reproducibility (24).

16 downregulated and 64 upregulated proteins were found in the aged sperm proteome compared to the young group. These differential proteins were enriched in various pathways, including protein digestion and absorption, galactose metabolism, pentose phosphate pathway, ubiquitin-mediated proteolysis, oxidative phosphorylation, glutathione metabolism, Parkinson’s disease, regulation of actin cytoskeleton, cholesterol metabolism and inositol phosphate metabolism. It has been established that clusterin and semenogelin-2, two members of the epididymal protease inhibitor (Eppin), are present on the human sperm surface to inhibit motility (25, 26). Clusterin expression is reportedly upregulated in many human diseases, including Alzheimer’s, type 2 diabetes and prostate cancer (27). Consistently, we found that sperm clusterin was 4.96-fold higher in aged males than in the young group. The decreased expression of Cilia and flagella-associated protein 44 (Cfap44) in the aged group may directly affect β-tubulin cleavage, preventing microtubule formation and eventually resulting in the decline of sperm motility (28). We also found that testis-specific H1 histone was downregulated expression in the aged group, which might be related to the lack of histone to protamine exchange, and eventually influence offspring through epigenetic effects (29).

UHPLC-Q-TOF/MS was adopted during metabolomic analysis since it can reportedly overcome the shortcomings of ultraviolet detectors and is adequate for analyzing the components of seminal plasma (30). 129 differential (62 downregulated and 67 upregulated) metabolites were found in the aged group and were enriched in 10 KEGG pathways, including citrate cycle (TCA cycle), glutathione metabolism, protein digestion and absorption pathways, Parkinson’s disease, glycerophospholipid metabolism, amino acid metabolism, oxidative phosphorylation, amino sugar and nucleotide sugar metabolism as well as cholesterol metabolism. Among these, citrate, creatinine, phosphoric acid, oxidized glutathione, and carnitine have been associated with sperm energy metabolism, oxidative stress, and sperm membrane damage. In addition, spermatogenesis and sperm function are highly energy-dependent, and low sperm ATP correlates with decreased sperm motility and fertilization rate (31). The decline of citrate (the first product of the TCA cycle involved in aerobic metabolism) in seminal plasma has been related to oligozoospermia (14). Oxidative phosphorylation is the primary energy metabolism pathway that mediates sperm motility (32). In the present study, citrate and oxidative phosphorylation were downregulated in the aged group, suggesting that the synthesis of ATP was reduced. In addition, carnitine is highly expressed in the epididymis and has been established to be essential for fatty acid metabolism in sperms. In this respect, lower levels of carnitine have been reported in the seminal plasma of infertile patients, and a strong positive correlation between L-Carnitine expression and sperm motility (33). Our study also found that the aged group had lower levels of Linoleoylcarnitine and Oleoyl-l-carnitine than the young group, indicating that fatty acid metabolism and energy production were reduced. Current evidence suggests the detrimental effect of oxidative stress on male fertility is mediated by damage to the sperm plasma membrane and DNA integrity (34). Reduced glutathione (GSH) and oxidized glutathione (GSSG), two predominant forms of antioxidants that are critical for protecting the cells from oxidative stress, are reportedly upregulated in the aged group (35). Besides, the GSH/GSSG ratio, an indicator of cellular health, has been reported to decrease in neurodegenerative diseases, such as Parkinson’s disease and Alzheimer’s disease (36). There is a rich literature available suggesting that glutathione could protect sperm from oxidative stress in semen, and glutathione supplementation benefits sperm morphology and motility (37). In addition, carnitine can protect sperm from oxidative damage and reduce spermatogenic cell apoptosis (38).

In addition to revealing possible mechanisms of decreased sperm motility and increased DFI in aged sperm, we established a biomarker panel that could identify aging semen through an integrated machine-learning method. Compared to one-dimensional statistical methods, machine learning is suitable for multi-dimensional variable analysis and can also select target molecules with high correlation, especially in constructing biomarker models to predict new samples. Furthermore, integrated machine-learning methods and integrating multiple machine learning algorithms were used to screen the most accurate biomarkers. In this study, four metabolites (Pipamperone, 2,2-Bis(hydroxymethyl)-2,2’,2’’-nitrilotriethanol, Arg-Pro, Triethyl phosphate) were used to construct an optimal biomarker panel and these four metabolic biomarkers were used to characterize aging semen. A simultaneous increase in these four metabolites indicates semen aging and poor sperm function, irrespective of age. This finding could help screen young males presenting with aging semen phenotypes and provide valuable insights to improve the fertility of such patients. However, a limitation of this study is the relatively small sample size used for the integrated machine-learning analysis. Indeed, a larger sample size is warranted to verify the accuracy and robustness of our biomarker panel for aging semen.

In conclusion, we corroborated that semen quality is related to male age and screened the differential metabolites and proteins between aged and young males, explaining the decline of semen quality in aged males to a certain extent. Besides, a biomarker panel was constructed to define aging semen. Our data improves currently available omics information on semen and provides the foothold for discovering novel biomarkers that can identify susceptible populations and improve semen quality and fertility of aged males.

## Data availability statement

The datasets presented in this study can be found in online repositories. The names of the repository/repositories and accession number(s) can be found in the article/**Supplementary Material**.

## Ethics statement

The studies involving human participants were reviewed and approved by Shanghai First Maternity and Infant Hospital’s medical ethics committee. The patients/participants provided their written informed consent to participate in this study.

## Author contributions

YG, XT and XJ conceived and designed the study. YY and HY contributed to the recruitment and characterization of the individuals. JL, QC and PK contributed to the sample collection, and FH contributed to analyze the data. YG and JL performed the experiments and wrote the manuscript. XT and WL critically revised the manuscript. All authors provided a critical review and approved the final manuscript.

## Supplementary material

Supplementary Figure 1 The correlation analysis of the candidate biomarkers.
