# Supplementary figures and images for: A new perspective on semen quality of aged male: The characteristics of metabolomics and proteomics
Source: Front Endocrinol (Lausanne). 2023 Jan 4;13:1058250. doi: 10.3389/fendo.2022.1058250 (PMC9848653; doi:10.3389/fendo.2022.1058250)

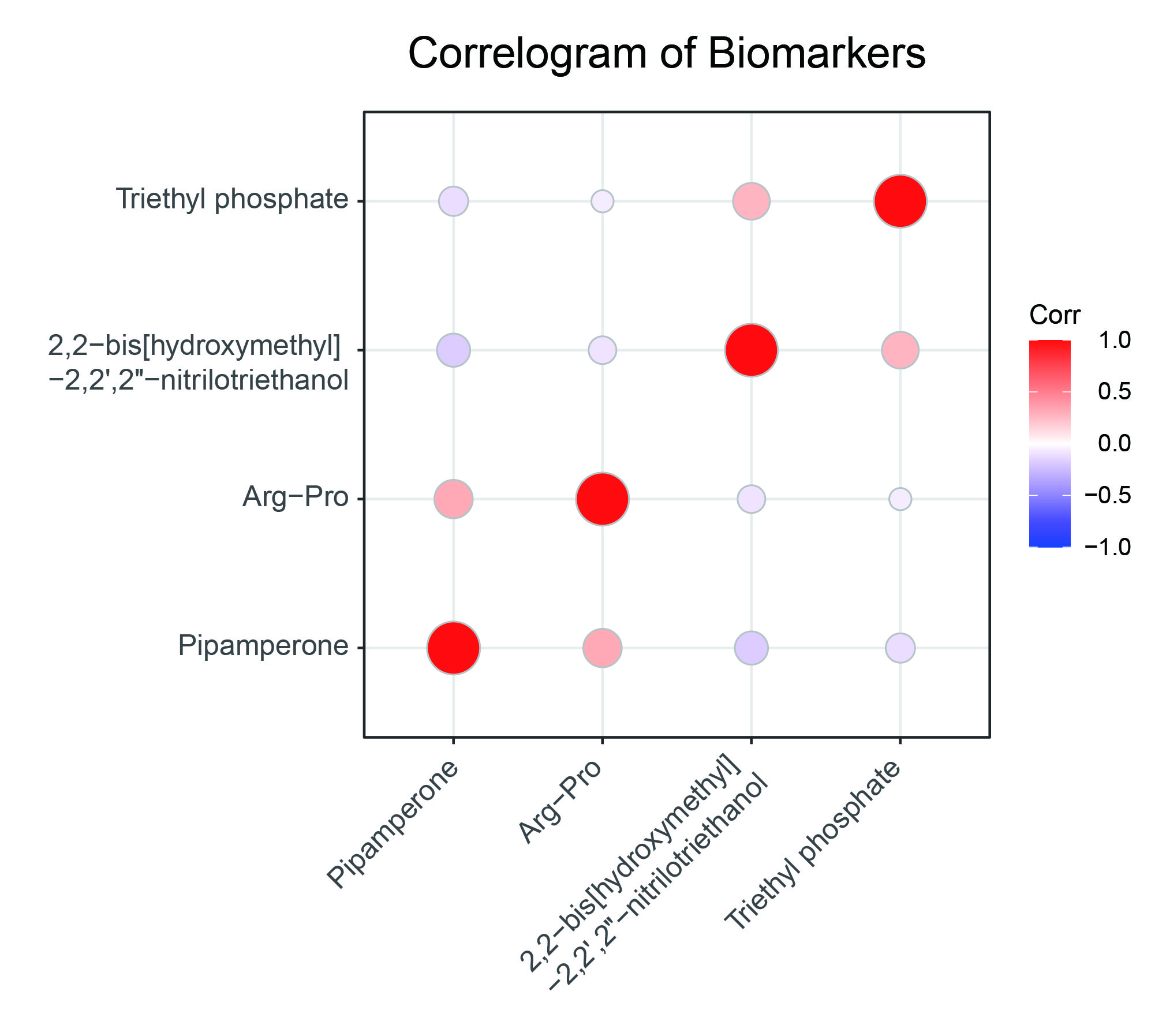

Supplement: Supplementary Figure 1 — The correlation analysis of the candidate biomarkers. [file Image_1.jpg]
