# Supplementary material for: A new perspective on semen quality of aged male: The characteristics of metabolomics and proteomics
Source: Front Endocrinol (Lausanne). 2023 Jan 4;13:1058250. doi: 10.3389/fendo.2022.1058250 (PMC9848653; doi:10.3389/fendo.2022.1058250)
Supplement: Supplementary file 2 [file Table_1.docx]

Table I. The descriptive statistics of semen parameters and DFI by male age category

| Age group | A(≤30) | B (31-35) | C (36-40) | D (41-45) | E(≥46) |
| --- | --- | --- | --- | --- | --- |
| Number (%) | 4638(36.16) | 5020(39.14) | 2094(16.33) | 744(5.80) | 329(2.57) |
| Abstinence time(days) | 4.21±2.87 | 4.29±2.88 | 4.49±3.24 | 4.71±4.40 | 4.88±3.29 |
| pH | 7.22±0.18 | 7.22±0.18 | 7.23±0.19 | 7.23±0.18 | 7.22±0.20 |
| Volume(ml) | 4.12±1.62 | 3.96±1.55 | 3.79±1.64 | 3.52±1.50 | 3.24±1.66 |
| Sperm concentration(10^6^/ml) | 23.75±17.51 | 23.79±17.96 | 25.55±19.28 | 25.67±19.98 | 30.16±25.57 |
| Total sperm count (10^6^) | 94.35±75.59 | 90.84±73.78 | 92.19±79.98 | 86.63±77.74 | 86.14±74.08 |
| Progressive motility (%) | 34.07±16.96 | 32.15±16.83 | 30.06±17.03 | 26.95±16.98 | 23.03±16.51 |
| Progressive motile sperm count (10^6^) | 35.27±38.18 | 32.03±35.63 | 30.02±36.00 | 25.12±28.76 | 22.32±27.21 |
| DFI (%) | 10.24±8.75 | 11.94±10.12 | 14.62±11.43 | 17.79±12.88 | 21.82±15.05 |

Data are expressed as mean±standard deviation. DFI DNA fragmentation index.

Table II. Linear regression analyses of age trend in semen parameters.

| Semen parameter | Β (95% CI) | SE | t | P value |
| --- | --- | --- | --- | --- |
| Volume (ml) | -0.04 (-0.05, -0.04) | 0.00 | -15.44 | <0.001 |
| Sperm concentration (10^6^/ml) | 0.18 (0.11, 0.24) | 0.03 | 5.70 | <0.001 |
| Total sperm number (10^6^) | -0.56 (-0.81, -0.32) | 0.12 | -4.55 | <0.001 |
| Progressive motility (%) | -0.46 (-0.52, -0.41) | 0.03 | -16.17 | <0.001 |
| Progressive motile sperm count (10^6^) | -0.65 (-0.77, -0.53) | 0.06 | -10.64 | <0.001 |
| DFI(%) | 0.49 (0.46, 0.53) | 0.17 | 28.78 | <0.001 |

*Adjustment for abstinence time and pH. CI: Confidence Interval. SE: Standard Error. DFI: DNA Fragmentation Index.
